# Prevention of childhood leukaemia by lifestyle changes

**DOI:** 10.1038/s41375-021-01220-6

**Published:** 2021-04-08

**Authors:** Christine J. Harrison

**Affiliations:** Translational and Clinical Research Institute, Newcastle University Centre for Cancer, Newcastle-upon-Tyne, England

**Keywords:** Medical research, Cancer prevention

In this Perspective [1], Greaves et al. pose the ambitious question: can childhood leukaemia be prevented? This research group has made significant contributions over the last decades in terms of understanding the processes of initiation and progression of childhood leukaemia, based largely on detailed investigations of certain subgroups of childhood acute lymphoblastic leukaemia (ALL) [2], thus they are in the strongest position to convince us of the possibility.

Childhood ALL is one of the greatest success stories of modern medicine: as a result of improved patient management and risk-adapted administration of established drug regimens, more than 90% of children now survive the disease [3]. Although astounding, the price paid for this achievement is prolonged and distressing therapies, with high levels of unpleasant side effects and lasting morbidities. Thus, prevention would provide an extremely welcome alternative.

Before considering prevention, those underlying causes of ALL, which may respond to intervention, need to be identified. The most popular explanations suggest that childhood ALL arises from an abnormal immune response to common infections, attributed alternatively to population mixing [4] or, as proposed by Greaves et al., delayed infection. The latter is based on normal functional development of the immune system during the perinatal period by exposure to microbial infection. This in turn, influences the composition of the infant microbiome, impacting on the health of the child [2]. Inadequate exposure to natural microbes distorts the immune system, which in infancy is predicted to dysregulate the response to common infections that could lead to ALL.

A two hit model of childhood ALL was verified by Greaves and co-workers from very elegant studies involving comparative genomics of concordant ALL in monozygotic twins compared to observations of discordant development of the disease in some twin pairs and back tracking of leukaemic clones to birth from the study of neo-natal blood spots [2]. Collectively these data have demonstrated that the majority of cases of childhood leukaemia are prenatal in origin [5].

From their understanding of the natural history of ALL, it develops in two critical steps: 1) an initiating event occurring in utero and 2) a postnatal mutational event that promotes development of overt leukaemia. Although multiple reported initiating mechanisms, involving endogenous oxidative stress, infections and inflammatory responses, further complicate the process, they are linked by activation of AID (activation-induced cytidine deaminase) within the pre-leukaemic stem cells. Then an abnormal immune response to infection(s) triggers the requisite secondary mutational events. Thus, it appears that common infections have two opposite effects on the risk of developing ALL, depending upon timing: antagonistic (early) or promotional (late). Indeed, mouse studies have provided evidence that delayed common infections can play a role in promotion of ALL [6] and that early stimulation of the immune system can be protective.

If we consider from these observations that most cases of childhood ALL may be preventable, it should be advertised as such by wide-scale promotion of beneficial yet modifiable lifestyle changes, including early social mixing and extended breast-feeding. The rationale is that these factors influence the way in which infants acquire their gut microbiomes, suggesting that the key risk factor for ALL in early life may emerge from the components of the gut microbiomes acquired around the time of birth, with lasting effects on immune function. How this mechanism may function is elegantly described within the Perspective.

In order to progress further, more evidence of deficient microbiomes in ALL, from extensive monitoring of infants, is required. Validation studies are underway within the Greaves’ group to demonstrate that microbiome booting can prevent infection promoted ALL within an animal model. However, many questions remain to be answered: what bacterial mix might be effective; how might it be safely delivered; how do we identify individuals at risk of ALL, who would receive such potentially protective treatment in infancy; or should it be available to all?

Although the focus here is on ALL, there is also evidence of gut microbiome dysregulation in other conditions, including childhood allergies, type 1 diabetes and possibly other autoimmune diseases, such as multiple sclerosis. Thus, promoting the benefits of specific life style changes should also highlight the potential for prevention of these conditions. In fact, the possible ramifications of microbiome dysbiosis for both childhood and adult health are far reaching.

Overall, this Perspective [1] presents credible preliminary evidence that gut microbiome boosting by simple life style changes or dietary supplements might present viable strategies for risk reduction or prevention of childhood ALL and potentially other serious diseases; the Greaves’ group have the ability to convincingly persuade us.
